# Harnessing spin effects for heterogeneous single-atom spin catalysis

**DOI:** 10.1093/nsr/nwae217

**Published:** 2024-06-22

**Authors:** Xu Han, Jinxing Chen, Peng He, Jiong Lu

**Affiliations:** Department of Chemistry, National University of Singapore, Singapore; Department of Chemistry, National University of Singapore, Singapore; Department of Chemistry, National University of Singapore, Singapore; Department of Chemistry, National University of Singapore, Singapore

## Abstract

This perspective explores detailed structural design and strategies for spin regulation in single-atom spin catalysis, enabling unparalleled efficiency in chemical transformations through the harnessing of spin effects combined with atomic precision of active sites.

Single-atom catalysts (SACs) are renowned for their remarkable efficiency and precise atomic utilization, offering meticulous control over the atomic environment and electronic structures of well-defined active centers. This level of control holds the promise of achieving unparalleled catalytic performance and selectivity. However, maintaining high efficiency during spin-flip catalytic processes necessitates the design of SACs with spin-active sites, known as heterogeneous single-atom spin catalysts (SASCs) [1,2]. Spin catalysis, proposed two decades ago, typically refers to chemical reactions influenced by or related to the electron spin of reactants or catalysts, also known as spin-responsive catalysis [3]. Conventional spin catalysts (SCs), typically consisting of homogenous transition-metal compounds, alter their spin multiplicity through spin-crossover to lower the reaction barrier or alter the reaction pathway [3]. SCs responsible for modulating electron angular momenta (spins) of the reactants, play a pivotal role in facilitating the transition of inert reactants into spin-allowed states. This enables the effective surmounting of the spin-forbidden reaction barrier, thereby promoting spin-related catalytic processes. In contrast, heterogeneous SASCs, typically featuring structurally well-defined spin-active centers, involve the immobilization of individual metal atoms, often magnetic dopants, on solid supports such as carbon-based materials [4–6], metal oxides [7] or transition-metal dichalcogenides (TMDs) [1,2], to serve as active sites for catalytic reactions (Fig. 1a). SASCs combine the distinctive characteristics of traditional SACs with the adjustable spin effects [3], which can excel in spin-related catalytic reactions featuring divergent spin states between reactants and products.

**Figure 1.**
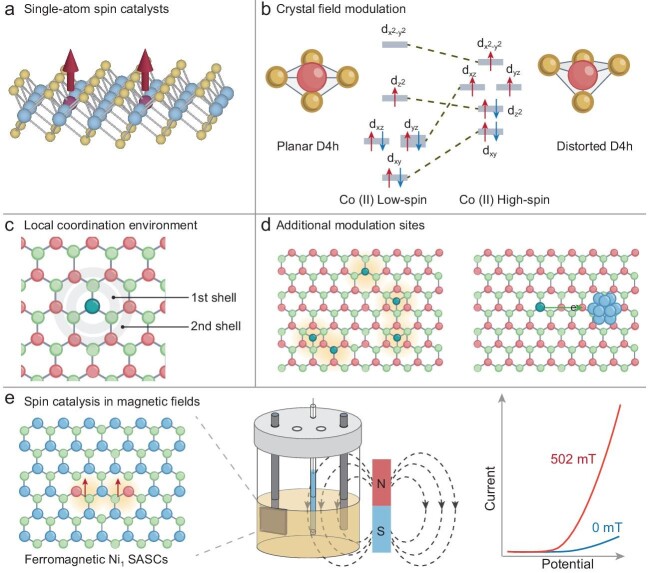
Schematic illustrating the spin regulation methods of SASCs: (a) illustration of single-atom spin catalysts with individual magnetic metal atoms on heterogeneous support, and spin regulation through (b) manipulating the crystal field, (c) tuning the local coordination environment at active sites, (d) introducing additional modulation sites by doping single-atoms and particles adjacent to the active sites and (e) applying an external magnetic field on ferromagnetic SASCs.

The spin state of active sites in SASCs can be manipulated to achieve optimal catalytic activity and selectivity. The common strategies for the spin manipulation of these SACS involve (i) manipulating the crystal field at active sites *via* altering their symmetries (Fig. 1b), (ii) tuning the local coordination environment (Fig. 1c), (iii) introducing additional modulation sites (eg. single-atom and particle sites) adjacent to the active sites (Fig. 1d) and (iv) applying an external magnetic field (MF) (Fig. 1e).

From the perspective of crystal field regulation, altering molecular conformations to induce symmetry change of active sites can effectively tune the orbital energy and degeneracy, leading to rearrangements in electron filling and resulting in different spin states. For instance, lowering the symmetry of coordination configurations of single-atom metal sites often results in alterations in orbital energies and potentially an increased number of unpaired *d*-electrons, thereby facilitating the generation of a high-spin (HS) state (Fig. 1b) [4]. The experimental implementation of this strategy involves thermal treatment to create more energetically favorable non-planar sites [4], or substitutional replacement of metal atoms in TMDs with single magnetic dopant to create non-centrosymmetric spin centers [1], thereby inducing an HS state. The resulting HS state of SASCs with partially occupied orbitals renders the electrons more delocalized and enhances the orbital overlap with adsorbates. This increases the potential to facilitate the injection of spin-polarized electrons injected into a spin-flipping intermediate and also enhance electron backdonation to weaken the bond order. Consequently, this promotes spin state transitions of intermediates and simultaneously decreases the reaction energy barrier more effectively than the low spin (LS) state [4,6].

Tuning the coordination environment from both the first and second coordination shells of metal atoms in SACS proves to be an effective method for optimizing the spin states of active sites (Fig. 1c). For instance, the introduction of oxygen vacancies [7], perpendicular ligands [8], or changing the type of coordinated atom [9] can all influence the transition from an LS state of the metal to an intermediate spin (MS) or HS state by either donating or withdrawing electron density through direct bonding with the central metal. For example, a significant orbital overlap between the central metal and the delocalized ligand orbitals enables direct electron transfer between them [9]. The dynamic coordination of SACs also results in local spin transition between HS and LS that reduces the reaction barrier through spin-crossover of the active site [8]. The resulting MS or HS sites can effectively donate electrons to the π* orbital of the triplet state *O_2_ or doublet state *NO, modulating orbital overlap and promoting ferromagnetic coupling of intermediates to enhance spin exchange. Consequently, this reduces the spin-flip barrier for forming the singlet state OH^−^/H_2_O or *NHO to accelerate spin non-conservation reactions [7,8].

Introducing additional modulation sites, such as single-atoms [2,6] and particles [5] adjacent to active sites, enables spin regulation from a relatively remote distance through electron redistribution (Fig. 1d). This can be facilitated by the strong metal-support interactions and the use of highly conductive supports that enhance electronic interactions between active sites and modulation sites, promoting the LS-to-HS transition of the central metal atoms [5]. The electron redistribution between active sites and modulation sites can also induce band broadening to narrow the bandgap, thus promoting spin-polarized electron transfer [9]. Long-range spin exchange interactions between doped magnetic single atom sites can be tuned to favor ferromagnetic coupling, which can in turn influence the spin density of active sites. The local configuration and spatial arrangements of single-atom modulation sites also have a great impact on spin-regulation of nearby active sites. Our recent work reports an adjacent Co_Ta_ site (substitution of Ta by Co in TaS_2_) and a hollow Co_HS_ site can both enhance the spin density at the Co_HS_ site, with an optimal enhancement for the Co_Ta_ site but an excessive enhancement for the Co_HS_ site. Thus having Co_Ta_ modulation sites optimizes the binding strength of O• over the Co_HS_ site to accelerate the oxygen evolution reaction (OER) [7].

In addition, the introduction of an MF can also affect the spin orientation and domain alignments typically in ferromagnetic SASCs. As a result, it can modify the spin density of active sites to optimize the adsorption energy of reaction intermediate species. Our recent work reports the fabrication of ferromagnetic SASCs through the substitutional replacement of Mo sites with a Ni single-atom to form a distorted NiS_4_ active center in a MoS_2_ host [1]. This substitution results in an unpaired 3*d* electron of Ni dopant site, creating the local magnetic moment associated with individual NiS_4_ sites. At high loadings, when the spacing between active sites is reduced, magnetic exchange interactions between adjacent NiS_4_ sites favor the long-range ferromagnetic ordering. Upon application of a mild *B* field, the optimization of spin density over the exposed sulfur (S) active sites can be enhanced as more ferromagnetically coupled adjacent sites can be created during domain realignment [1]. This favors the formation of more parallel spin configurations among neighboring S sites, thereby facilitating efficient generation of triplet dioxygen (↑O = O↑) during OER (Fig. 1e). Additionally, MF can also induce Zeeman splitting and induce the spin transition from LS to HS, enable changes in spin multiplicity of the system, thus enhancing the spin-related reactions [6].

In summary, SASCs featuring atomically dispersed spin-polarized active sites with precisely regulated spin states can demonstrate exceptional spin catalytic performance. Beyond the extensively explored electrocatalytic applications, SASCs hold promise for advancing chemical transformations between different spin states of radicals or intermediates, such as cyclization, cycle opening, or coupling reactions. Furthermore, heterogeneous geminal atom catalysts (GACs) represent an emerging class of catalytic solids with pairs of dynamic single-atom sites for efficient cross-coupling reactions [10]. Designing a GAC with spin-polarized sites could be an exciting direction to accelerate spin-selective chemical transformations by combining the benefits of dynamic atomic-precision catalysis and spin catalysis.
